# Supplementary material for: Dose-response relationship of photobiomodulation therapy and oxidative stress markers in healing dynamics of diabetic neuropathic ulcers in Wistar rats
Source: J Diabetes Metab Disord. 2022 Dec 22;22(1):393–400. doi: 10.1007/s40200-022-01157-2 (PMC10225440; doi:10.1007/s40200-022-01157-2)
Supplement: Supplementary file 1 — Supplementary Material 1 [file 40200_2022_1157_MOESM1_ESM.pdf]

Gagana

by Gagana Gagana

Submission date: 30-Jul-2022 03:08PM (UTC+0800)
Submission ID: 1876831853
File name: Gagana_Manuscript.docx (23.21K)
Word count: 2650
Character count: 14929

Introduction

Diabetic foot ulcers (DFUs) are reported to be one of the most expensive complications of diabetes mellitus (DM), with high morbidity and mortality rates [1]. Among patients with diabetes, approximate 20% of the hospital admissions are due to the appearance of DFUs. DFU is a localized injury to the underlying tissue or skin below the ankle [2]. DFUs are of multifactorial origin, and factors like uncontrolled hyperglycemia, chronic diabetic peripheral neuropathy (DPN), and peripheral vascular diseases (PVD) play a central role in the cause of DFUs [3].

The global prevalence of DFUs is found to be 7-8%. The incidence is higher in older patients and much lower in young adults. In India, a community survey in rural Udupi, Karnataka, has reported the prevalence of DFU to be 6.38% in people with type 2 diabetes mellitus (T2DM) [4]. The treatment of DFUs is a formidable clinical challenge because these wounds are characterized by delayed healing, often becoming chronic. Therefore, the treatment of DFUs should be effective and successfully relieve the direct and indirect cost burdens for the patients [5].

Currently, in clinical practice, several approaches address the challenges associated with diabetic wound healing, including surgical, pharmacological, conservative and non-pharmacological modalities [6, 7]. Among which a promising non-pharmacological approach is Photobiomodulation therapy (PBMT). Non-ionizing light sources are employed in PBMT. These light sources include LEDs, LASERs, etc. The principle of PBMT is that it causes low-energy bio-stimulation and stimulates the photochemical reactions at the injured site (cells/ tissue) [8]. PBMT is found to have beneficial therapeutic outcomes, including immune modulation, pain control, stimulation of tissue growth and wound healing [9]

Although PBMT is used in clinical practice to treat varieties of diseases, its application is still a controversial topic. The reason is its underlying biochemical reaction, and the mechanism of action in the body is unclear. The parameters of PBMT, such as the wavelength, irradiation time, dosage, power- density, and pulse type, must be optimized for each treatment [10].

Therefore there is a strong need to establish the PBMT response relationship that could describe the magnitude of the response of the diabetic wound condition as a function of stimulus after exposure of PBMT to different wavelengths, dosage and time. The present study aims to establish the PBMT response to varying doses of 4-15J/cm^2 during the healing of diabetic neuropathic ulcers in relation to the oxidative stress markers of wound healing.

Materials and Methods

Ethical Clearance

We sought ethical clearance from the "Institutional Animal Ethics Committee" from KMC, MAHE- Manipal. Ethical clearance number: IAEC/KMC/95/2018

Animal selection and care

A total of 126 female Albino Wistar rats were procured. (Mean bodyweight 220.62±11.69 g and a mean age of 5.43±0.11 months). The standard laboratory environment for the animals was maintained.

Induction of diabetes and confirmation

We induced diabetes in all animals using 1 mL intraperitoneal injection of streptozotocin (60mg per kg of body weight) prepared with 0.1M citrate buffer (pH 4.5). Animals were placed in a separate metabolic cage for seven days. Fasting blood- glucose was measured for each animal (Glucose oxidase peroxidase method). Animals with blood-glucose levels ≥200 mg/dL were included in the study.

Neuropathy induced by sciatic nerve damage and confirmation

Neuropathy was induced by crushing the sciatic nerve of the left/right hind leg. Before the procedure, animals were anaesthetized with intravenous ketamine. The hind leg was shaved using a blade. The sciatic nerve was demonstrated until the mid-thigh level under sterile conditions. Using the watchman's forceps tip, the sciatic nerve was crushed for 10 seconds (2×15s). The animals were observed for three weeks of neuropathy's behavioural and clinical modifications.

We performed confirmatory tests for neuropathy using the hind-paw withdrawal test for hot and cold stimulus and response to 10g monofilament for paw withdrawal. The response to a stimulus within 5 seconds was considered normal. And more than 5 seconds was considered a delayed response in the neuropathy-induced leg.

Excisional wound model

After the confirmation of neuropathy, an excisional wound ($2cm^2$) on the femur of the neuropathic leg was created. Each animal was maintained in a separate cage.

Grouping of Animals

The animals were divided into six experimental-groups and one control-group based on similar body weight and blood-glucose levels. **(Figure-1)**

Photobiomodulation therapy protocol

PBMT irradiation method was standardized before the experiment. We have used PBMT of scanning laser wavelength 655 nm (Visible red) of continuous wave emission and power output 24 mW and power density 2.46 mW/cm^2 and spot size 9.1, a probe laser of wavelength 808 nm

(Infra-red) of continuous wave emission and power output of 120 mW and power density 120 mW/cm^2 and spot size 1 cm^2 were used.

We treated the experimental-group with PBMT of dosages 4, 6, 8,10,12,15 J/cm^2 to establish the dose-response relationship in wound healing dynamics. The PBMT irradiation time was between 3 minutes to 12 minutes, depending on the doses. The average duration of PBMT treatment in the experimental groups lasted from 14 to 20 days across the group. The control group animals did not receive any PBMT.

Mean wound healing and wound contraction

The area of the excisional wound was tracked and documented on a transparent white sheet. The traced wound area was measured in centimetres using the Smith and Jones wound area measurement graph and represented in cm^2. The wound contraction rate was calculated using the formula "Initial area (I)-final area (F)/the number of days (N)" and represented in per day cm^2.

Biochemical Analysis

We used the spectrophotometric method to determine Malondialdehyde (MDA) levels (Kei Satoh's Method) in the present study. The concentration of MDA was represented in nmol/ml. Superoxide dismutase (SOD) was assayed according to the spectrophotometric determination method of Kakkar et al. The levels of SOD were represented in nmol/ mg of protein. Reduced glutathione (GSH) was estimated using spectrophotometric determination by Ellman et al. method. The tissue GSH levels were represented in mmol/mg of protein.

Statistical Analysis

Statistical analysis was done using EZR (R version 3.4.1) software, and findings were presented in Mean ± SD. One-way ANOVA with post hoc Dunnett's test was used for many to-one comparisons. The level of significance was kept to be $p<0.05$. The effect size was calculated to compare the dose-response.

Results

Rate of contraction of wound

According to the results of our study, the experimental group receiving 4J, 6J, and 8J treatments had a wound closure rate that was better and quicker than the control group with mean values of (0.291±0.009), (0.316±0.10), and (0.129±0.001) respectively (p-value <0.05). However, the experimental group treated with 10J, 12J, and 15J/cm^2 showed a delayed rate of wound contraction with mean values (0.11±0.005), (0.07±0.03), (0.07±0.05), respectively. **(Figure-2)**

Biochemical Analysis

The oxidative markers help in a better understanding of the oxidative status of the wound. We evaluated the tissue MDA, SOD, and GSH levels considered among the potent markers in the present study. **(Figure-3)**

Tissue MDA levels

MDA represents the lipid peroxidation occurring at the wound site. The increased MDA represents the increased peroxidation. The present study found that MDA levels increased significantly in all the experimental groups during the inflammatory phase than in the control group ($p<0.05$). However, during the proliferative phase, MDA levels decreased significantly in the experimental group treated with 6J/cm^2 and 8J/cm^2 ($p<0.05$) **(Figure 3A)**. The

experimental group treated with 8J/cm^2 showed a strong dose-response relationship with an effect size of 2.95.

Tissue SOD Levels

Superoxide dismutase catalyzes the dismutation of superoxide radicals into molecular hydrogen and hydrogen peroxide. SOD has a potent anti-inflammatory activity. In the present study, during the inflammatory phase, we observed an increased SOD activity in all the experimental groups compared to the control ($p<0.05$). However, the experimental groups treated with 4J and 6J showed significantly higher SOD levels during the remodelling phase ($p<0.05$) **(Figure 3B)**. The effect size was 5.30, with a strong dose-response relationship in the experimental group treated with 6J/cm^2

Tissue GSH levels

Reduced glutathione is the antioxidant present in almost all aerobic organisms. They are capable of preventing the cellular damage produced by free radicals. In the current study, we saw elevated tissue GSH levels throughout the wound-healing phases in all the experimental groups. These changes were significant in the PBMT groups treated with 4, 6 and 8J/cm^2 **(Figure 3C).** However, the dose-response was strong in the 4J/cm^2 group, with an effect size of 5.4.

Discussion

Diabetes and delayed wound healing

DM When left untreated for a prolonged period, persistent hyperglycemia causes the blood vessels to accumulate sugar complexes, which causes poor blood flow and aberrant nerve functioning. In our study, we have demonstrated the diabetic neuropathic wound

condition to assess PBMT's effectiveness in treating the diabetic neuropathic wound. PBMT has significantly improved wound healing dynamics as wound treatment has progressed. The *In vitro* and *in vivo* studies have revealed that the optimal PBMT promotes cell motility, viability, and proliferation of diabetic cells [9]. Our findings indicate that compared to the control group, the experimental group of animals given PBMT at dosages of 4J, 6J, and 8J had a significantly faster rate of wound contraction and a quicker mean wound healing time. However, PBMT of dosages 10J, 12J, and 15J demonstrated a slower rate of wound contraction **(Figure-2)**.

PBMT in diabetic wound healing

It is possible that the group treated with PBMT of dosages 4-8J/cm2 showed better wound healing because the multi-step process of wound healing includes vascular and cellular changes that result in epithelial regeneration, fibroblasts proliferation, collagen formation, revascularization and wound contraction. The non-thermal effects of PBMT deliver the light energy directly to the cells, and the photon in the excited state stimulates the cellular metabolism. The energy absorbed by the photo acceptor can be transmitted to other molecules, triggering bio stimulatory processes in the surrounding tissue and noticeable changes at the wounded site [11].

It is claimed that PBMT can lead to increased mitochondrial activity with the consequent increase in the production of ATP, protein synthesis, cellular migration, proliferation and neo-angiogenesis [12]. Neovascularization, epithelialization, and the formation of granulation tissue are characteristics of biostimulation of PBMT, which promotes cell proliferation in post-inflammatory stages [13]. Another possibility is that neo-angiogenesis is enhanced or upregulated by PBMT, increasing blood flow surrounding the wound. Additionally, PBMT's

ability to inhibit potentially detrimental reactions during the inflammatory phase, promoting collagen production, may have aided wound healing [14, 15].

These findings are similar to the previous studies in the literature where they have highlighted that the therapeutic dose of PBMT of dosage 3-6 J/cm^2 has accelerated the wound healing, whereas PBMT of higher dosages decelerated the wound healing process. An in vivo study by Al- watban et al. in 2001 found that the wounded cells irradiated with He-Ne laser of 638 nm wavelength and 5J dosage showed increased activity of mitochondria, increased fibroblastic proliferation as well as improved microcirculation [16]. In 2008, channual et al. found that the open skin wound of the rats treated with 585nm and 7J/cm^2 showed improved vascular proliferation [17].

Similarly, in 2009, Maiya et al. treated excision wound skin with He- Ne laser of 625 nm and found that the group treated laser of dosages 4-5J/cm^2 increased the production of granulation tissues [18]. A study by Hawkins et al. in 2006 studied the effect of PBMT on wounded human skin fibroblasts and found that the PBMT of dosages 10J and 16J showed a reduction in cell proliferation and migration [19]. Similarly, in 2007, Houreld N et al. found that the diabetic wounded fibroblast cells treated with 16J/cm^2 showed decreased cellular migration [20]. Therefore, it appears that PBMT is having a direct impact on the experimental groups treated with 4-8J as compared to the non-irradiated control group.

Oxidative stress in delayed wound healing

During cellular respiration, the oxidation of carbohydrates, lipid and protein molecules occurs with the complex molecules' enzymatic cleavage, resulting in the formation of singlet molecules called reactive oxygen species. Uncontrolled DM led to a rise in free radicals, low molecular antioxidants and depleted scavenging enzymes are being glycosylated, causing oxidative-stress and delayed healing [21].

Mitochondria and NADPH oxidase are the principal sources for the production of ROS. Through oxidative phosphorylation. The excessive ROS created causes oxidative damage. In the inflammatory phase, when ROS levels are accumulated at the wound lesion, it plays a harmful function by causing neutrophils and macrophages to generate high levels of ROS, as well as pro-inflammatory cytokines and proteolytic enzymes [22].

In normal wound healing, cells can produce antioxidants that scavenge ROS molecules. The SOD and catalase dismutases these ROS to hydrogen peroxide (H_2O_2) and molecular oxygen. In diabetic wound healing, the antioxidant enzymes are not generated in sufficient quantities to normalize the ROS effects. However, Due to the auto-oxidation of glucose, glycosylation of scavenging enzymes, and depletion of low molecular antioxidants, ROS levels will be beyond in diabetic wound conditions, delaying the healing process [23].

In our study, although tissue MDA-levels were increased during the inflammatory phases of healing, the MDA levels were decreased in the group treated with PBMT of doses 6J and 8J across the proliferative phase. We observed an effective increase in the tissue SOD levels in the experimental group treated with 4J and 6J during inflammatory and remodelling phases of healing. Similarly, the reduced glutathione levels were significantly increased in the experimental groups compared to the control **(Figure-3).** We have also observed s strong dose-response relationship between these markers and the experimental groups treated with 4J, 6J, and 8J compared to control and the other doses.

Measurement of MDA in the serum is a good reflection of free radical generation since the levels of MDA represent the redox reaction taking place, reflecting the free reactive oxygen species available for conjugation. The higher the single molecules, the more the compound formation.

We discovered an effective rise in the antioxidant enzyme of the experimental groups treated with PBMT compared to the unirradiated controls in the present research. In diabetic individuals, an increase in free radicals and decreased antioxidant activity may aggravate the condition and account for the delay in wound healing and closure.

Similar results were observed in Tatmatsu-Rocha JC et al. (2016), who used the super pulsed 904 nm laser at a dose of 2.39J/cm^2, and Denadai AS et al. 2017, who used PBMT 6J/cm^2 and 660 nm to treat diabetic skin lesions. The possible mechanism could be that the photo stimulatory effect of PBM on mitochondria accelerates the synthesis of deoxyribonucleic acid (DNA), Adenosine triphosphate (ATP) production, modulating ROS and nitric oxide (NO) productions [24, 25].

The mitochondrial redox potential of the electron transport chain is enhanced by the non-thermal photochemical reactions of the PBMT and sensed and transmitted to the cytosol to regulate catalase activity and other enzyme activations. Since antioxidant molecules can neutralize the effects of ROS, PBMT can mediate cell signalling to create them. It is clear that PBMT, a crucial factor in fibroblastic proliferation and angiogenesis in wound healing, facilitates the net quantity of ROS and antioxidants essential for healing wounds. [26].

Strength of the study

- To the best of our knowledge, this is the first study conducted in a diabetic neuropathic excisional wound model using oxidative markers in six different PBMT doses.
- The present study is the first study to establish the dose-response using clinical and biochemical outcomes.

Limitations of the study

- Total ROS and antioxidant levels have not been evaluated

Clinical implications

• In light of the results of the current investigation, we suggest that PBMT be considered as one of the promising adjuvant modalities in clinical practice.

• In view of our findings, the oxidative markers may provide a clear indication of the wound status to PBMT response.

Conclusion

- PBMT of dosages 4, 6, and 8J showed a better and faster healing rate in the diabetic neuropathic wound
- PBMT regulated the ROS and antioxidant levels with optimal changes in MDA, SOD and GSH and accelerated the healing process
- In addition, we found that PBMT showed a strong dose-response relationship in the groups treated with 4J, 6J and 8J

Gagana

ORIGINALITY REPORT

10%	9%	8%	%
SIMILARITY INDEX	INTERNET SOURCES	PUBLICATIONS	STUDENT PAPERS

PRIMARY SOURCES

1	www.ncbi.nlm.nih.gov<br>Internet Source	5%
2	Gagana Karkada, G. Arun Maiya, Praveen Arany, Mohandas Rao, Shalini Adiga, Shobha Ullas Kamath. "Effect of Photobiomodulation Therapy on Oxidative Stress Markers in Healing Dynamics of Diabetic Neuropathic Wounds in Wistar Rats", Cell Biochemistry and Biophysics, 2021<br>Publication	1%
3	ir.cput.ac.za<br>Internet Source	1%
4	Nicolette N. Houreld. "Cellular Damage in Diabetic Wounded Fibroblast Cells following Phototherapy at 632.8, 830, and 1064 nm", Laser Chemistry, 2007<br>Publication	1%
5	Bisht, S.S.. "A convenient synthesis of novel pyranosyl homo-C-nucleosides and their antidiabetic activities", Carbohydrate Research, 20110715	1%

Publication

6	Orkun Ilgen, Ismet Hortu, Gokay Ozceltik, Gurkan Yigitturk, Oytun Erbas, Nedim Karadadas. "The Effects Of Methylene Blue On Ovarian Torsion-Detorsion Injury In A Rat Model", Journal of Pediatric and Adolescent Gynecology, 2020<br>Publication	1%
7	bpspubs.pericles-prod.literatumonline.com<br>Internet Source	<1%
8	www.ptfarm.pl<br>Internet Source	<1%

Exclude quotes	On	Exclude matches	< 3 words
Exclude bibliography	On		
