# Supplementary material for: Dose-response relationship of photobiomodulation therapy and oxidative stress markers in healing dynamics of diabetic neuropathic ulcers in Wistar rats
Source: J Diabetes Metab Disord. 2022 Dec 22;22(1):393–400. doi: 10.1007/s40200-022-01157-2 (PMC10225440; doi:10.1007/s40200-022-01157-2)
Supplement: Supplementary file 2 — Supplementary Material 2 [file 40200_2022_1157_MOESM2_ESM.docx]

30 July 2022

To,

The Editor

Journal of Diabetes & Metabolic Disorders

**Dear Sir,**

**Subject: Re- submission of the revised manuscript for consideration for publication**

We are obliged to submit our revised manuscript for consideration for publication in your esteemed journal, “Journal of Diabetes & Metabolic Disorders”. The research article is an original work titled “Dose- response relationship of photobiomodulation therapy and oxidative stress markers in healing dynamics of diabetic foot ulcers in Wistar rats”.

The present research aims to determine the effect of photobiomodulation therapy, a promising non-pharmacological modality in the healing dynamics of diabetic wounds, with special emphasis on oxidative stress markers. The findings from the present study may add a solid piece of evidence to the existing literature on diabetes wound management.

Highlights of the study are:

- PBMT of dosages 4, 6 and 8J/cm^2^ enhanced the overall wound healing dynamics in the diabetic neuropathic ulcer
- PBMT accelerated the rate of wound contraction and reduction in the mean healing time required for wound closure compared to the un-irradiated wounds
- PBMT modulated the ROS and antioxidant levels and thereby improving the oxidative status of the wound

There was a strong dose-response relationship in the experimental groups treated with 4, 6 and 8J/ cm^2^ and oxidative markers of the wound

On behalf of all the contributors, I will act as guarantor and will correspond with the journal from this point onward. The work submitted is an original work conducted, is not published before, or it is not under consideration for publication anywhere else. Its publication has been approved by all co-authors, and the publisher will not be held legally responsible should there be any claims for compensation.

Yours’ sincerely,

Corresponding Author

Dr G Arun Maiya PhD

Professor and Dean

Department of Physiotherapy; Manipal College of Health Professions (MCHP)

Chief: Centre for Diabetic Foot Care and Research (CDFCR), Kasturba Hospital

Manipal Academy of Higher Education (MAHE) Manipal, Karnataka, India -576 104

Membership Director: World Association for photobiomoduLation therapy (WALT)

President: Indian Podiatry Association –Karnataka Chapter

Ph.: +91 9845350823; Email: [arun.maiya@manipal.edu](mailto:arun.maiya@manipal.edu) ; [arun.maiya.g@gmail.com](mailto:arun.maiya.g@gmail.com)
